# Transfer of naturally acquired specific passive immunity against *Anaplasma phagocytophilum* in foals in Southeastern Pennsylvania and Northern Maryland

**DOI:** 10.1111/jvim.16812

**Published:** 2023-07-28

**Authors:** Emily K. Rule, Ashley G. Boyle, Darko Stefanovski, Eman Anis, Jennifer Linton, Olivia Lorello

**Affiliations:** ^1^ Department of Clinical Studies, New Bolton Center University of Pennsylvania Kennett Square Pennsylvania USA; ^2^ Department of Pathobiology, New Bolton Center University of Pennsylvania Kennett Square Pennsylvania USA; ^3^ B.W. Furlong and Associates Califon New Jersey USA

**Keywords:** colostrum, equine granulocytic anaplasmosis, foal, IgG, maternal immunity

## Abstract

**Background:**

Equine granulocytic anaplasmosis (EGA) is a common disease in adult horses, but clinical disease in foals is rarely reported. The relationship between equine maternal and neonatal antibodies to *Anaplasma phagocytophilum* is unclear.

**Hypothesis/Objectives:**

That mares in an endemic region would be seropositive for *A. phagocytophilum* and that mare and foal serum IgG concentrations for *A. phagocytophilum* would correlate. Additionally, we hypothesized that foal IgG concentrations for *A. phagocytophilum* acquired by passive immunity would decline by 6 months of age.

**Animals:**

Twenty‐two healthy mare‐foal pairs.

**Methods:**

This prospective observational study investigated serum IgG concentrations specific for *A. phagocytophilum* in mares and foals using an immunofluorescent antibody test (IFA). The association between foal titer (as a binary variable) and age in months was assessed using a mixed‐effects logistic regression.

**Results:**

A positive correlation between newborn foal antibody titers and mare titers was identified at both the pre‐foaling (τ_a_ = 0.38, τ_b_ = 0.50, *P* = .009) and foaling timepoints (τ_a_ = 0.36, τ_b_ = 0.47, *P* = .01). In *A. phagocytophilum* seropositive neonates, it was unlikely that a positive titer would be detected by 3 months of age (OR = 0.002, *P* = .02, 95% CI: 0.00001‐0.38). Three out of 20 foals seroconverted between 3 and 6 months of age.

**Conclusions and Clinical Importance:**

Transfer of specific passive immunity to *A. phagocytophilum* occurred in 80% of foals born to seropositive mares and declined by 3 months of age. *A. phagocytophilum* infection should be considered in foals displaying clinical signs consistent with EGA.

**Abbreviations**
EGA: equine granulocytic anaplasmosis
IgG: immunoglobulin G

## 1 INTRODUCTION

Equine granulocytic anaplasmosis (EGA) has is a disease associated with adult horses,^1^, ^2^ and clinical disease in foals is rarely reported.^3^, ^4^ Horses <4 years of age with EGA display milder clinical signs^5^ and therefore might not be diagnosed. There are 2 reports of EGA in single foals in the literature,^3^, ^4^ and most textbooks associate EGA only with adult disease. *Anaplasma phagocytophilum*, the causative agent of EGA, is a tick‐borne, obligate‐intracellular, gram‐negative bacteria that resides primarily within neutrophils and eosinophils of infected animals.^6^, ^7^ The mechanisms that lead to control of *A. phagocytophilum* infection in equids are not completely understood, although naturally infected horses develop high titer of specific antibody which can persist for a year or longer after active infection.^2^ Reported seroprevalence in horses varies across regions, and in North America ranges from 0.53% to 10.4%.^8^, ^9^ In Pennsylvania, 44.3% of horses presenting with suspected tick‐borne disease had positive antibody titers to *A. phagocytophilum*.^10^ Additionally, 3% of humans in the Northeast United States have serologic evidence of exposure to the *Ehrlichia* genus, as *A. phagocytophilum* was classified until 2001.^11^, ^12^ Cross‐reactivity due to homology in the 16 rDNAs between *Ehrlichia equi* and *Ehrlichia phagocytophilum* resulted in the reclassification of these bacteria into a single species, *A. phagocytophilum*, in 2001.^12^

The relationship between maternal and neonatal antibodies to *A. phagocytophilum* is unclear. The purpose of this study was to investigate transfer of naturally acquired passive immunity to *A. phagocytophilum* from dams to foals born in an endemic region. We hypothesized that in an endemic region, antibodies against *A. phagocytophilum* would be present in pregnant mare serum and that the resulting foal's IgG concentration for *A. phagocytophilum* would correlate to mare IgG concentration. Adequate transfer of passive immunity from dam to foal could in part contribute to the low prevalence of the disease in foals. Additionally, we hypothesized that, in the foal, IgG concentration for *A. phagocytophilum* acquired by passive immunity would decline significantly by 6 months of age.

## 2 MATERIALS AND METHODS

### 2.1 Determination of sample size

Fisher z test power analysis determined that a sample size of 20 mare and foal pairs would be needed to determine a correlation between mare and foal *A. phagocytophilum* specific IgG titers (power of 80% and α ≤ 0.05; STATA 16.0). The *Null* hypothesis correlation was assumed to be non‐significant (*r* ≤ 0.35). For the correlation to be significant, we were expecting *r* ≥ 0.95. Thus, the expected magnitude of the change in correlation was δ = 0.6.

### 2.2 Sample collection and processing

Healthy mare and foal pairs living in Southeastern Pennsylvania or Northern Maryland were included. Four to 6 weeks prior to the expected foaling date, 10 mL of whole blood was collected from each mare and allowed to clot. Within 24 hours of foaling, a newborn examination was performed on the foal. Five and 10 mL of whole blood was collected from both the foal and mare, respectively, and allowed to clot. Three mL of heparinized whole blood was obtained from the foal for serum IgG to determine adequate passive transfer (defined as >800 mg/dL) using the point‐of‐care turbidimetric analyzer (POC‐TIA; Rapid DVM Test II, Value Diagnostics, MAI Animal Health, Elmwood, Wisconsin) at the New Bolton Center Clinical Laboratory the same day or via densimeter (591B Densimeter, Animal Reproduction Systems, Inc., Ontario, California) at the farm. At 3 and 6 months of age, 5 mL of whole blood was drawn from the foal and allowed to clot. Serum was separated from all samples and stored in 2 mL aliquots at −80°C. IgG concentration specific for *A. phagocytophilum* was measured in serum using an immunofluorescent antibody test (IFA) through the Pennsylvania Animal Diagnostic Laboratory, Harrisburg, PA through batch testing at the completion of sample collection. Seropositivity was determined at a value of 1:50 and then titrated to determine the end point dilution.

### 2.3 Statistical analysis

All analyses were conducted with Stata 17MP, StataCorp, State College TX, with 2‐sided tests of hypotheses and a *P* value <.05 as the criterion for statistical significance. Descriptive statistics were provided as frequency counts and percentages for categorical variables. Normality of the data was assessed by a Shapiro‐Wilk test. The association between mare titers and foal titers for all timepoints was assessed using Kendall rank correlation. The association between mare titers at birth and pre‐foaling was measured using Wilcoxon matched‐pairs signed‐rank test. The association between foal's titer (as a binary variable) and age in months was assessed using a mixed‐effects logistic regression, where the fixed effects were set as months as the categorical variable. Random effects were set on the level of individual mare. All associations reported as OR with their respective 95% CI.

## 3 RESULTS

Twenty‐two mare and foal pairs met the inclusion criteria of a healthy foal with adequate passive transfer. Breeds were as follows: 7 Thoroughbreds, 12 Standardbreds, 1 Warmblood, 1 Arabian and 1 Pony. Of the 22 mares, 19 (86%) had positive *A. phagocytophilum* titers at their pre‐foaling examination and 20 (91%) had positive *A. phagocytophilum* titers at the time of foaling. Foals born to seronegative mares (2/22) had negative antibody titers at all timepoints. Of the 20 mares that had positive titers at the time of foaling, 16 (80%) of their foals had positive titers at birth, and 1 foal had a missing sample. These data supported transfer of *A. phagocytophilum*‐specific antibodies from seropositive mares to healthy foals with adequate transfer of passive immunity (τ_a_ = 0.36, τ_b_ = 0.47, *P* = .01). There was no difference in titers for mares between the pre‐foaling and foaling timepoints (*P* = .5), and these 2 values were highly correlated (τ_a_ = 0.39, τ_b_ = 0.52, *P* = .006).

Blood was drawn from foals at an average of 102 days of age (±17 days) and 190 days of age (±19 days). Foals that did acquire *A. phagocytophilum* titers had a low likelihood of maintaining positive titers at 3 months (OR = 0.002, *P* = .02, 95% CI: 0.00001‐0.38) and 6 months of age (OR = 0.008, *P* = .03, 95%: 0.0001‐0.58). Of the 20 foals who were considered seronegative at 3 months of age, 3 foals (15%) had positive titers at 6 months of age. Two of the foals that seroconverted between 3 and 6 months of age demonstrated clinical signs of disease including fever (2) and intestinal hypermotility (1).

## 4 DISCUSSION

Seropositivity to *A. phagocytophilum* in clinically healthy pregnant adult mares at the time of parturition was 91% indicating high seroprevalence in the study region. Reported infection rates of *Ixodes scapularis* ticks with *Anaplasma* spp. in Pennsylvania are relatively low (1.7%‐3.3%).^13^, ^14^, ^15^ Complete medical records were not available for all mares, however no clinical signs of EGA were reported during the study period. In a recent study of horses with suspected tick‐borne illness in Pennsylvania, only 41.3% of horses had positive *A. phagocytophilum* antibody titers (≥1:50) on IFA assay.^10^ Seropositivity rates as high as 100% have been reported in apparently healthy horses from central New York and western New Jersey.^16^ Antibody response to acute infection with *A. phagocytophilum* in the horse takes several weeks,^17^ and the lower seropositivity rate in clinically affected horses could represent a risk factor for development of clinical EGA.

Due to the histologic structure of the mare's placenta, the foal is born with virtually no prenatal immunity and relies on colostrum for transfer of immunoglobulins, along with other cells and proteins, and establishment of a passive immune system. The colostral immunoglobulins, predominantly IgG in the equid,^18^ are selectively transferred from serum into the mammary gland.^19^ In the dam, this sequestration into the mammary gland might result in a decrease in circulating serum concentrations of immunoglobulins.^20^ When comparing serum titers of dams and calves, a higher percentage of calves were seropositive than dams, indicating that there might be a periparturient drop in serum IgG concentration in the dam.^21^ In the present study, there was no difference in mare titers between the pre‐foaling and foaling timepoints which did not support a decrease in IgG during colostrogenesis.

A positive correlation between newborn foal titers and mare titers was observed for both the pre‐foaling and foaling timepoints. In foals born to seropositive mares, 80% had positive titers to *A. phagocytophilum* at the day‐old examination. Similar correlations between mare and foal titers for West Nile Virus have been identified in ponies when adequate passive transfer is achieved.^22^

There is evidence of some transmission of maternal antibodies against *Anaplasma phagocytophilum* from mare to foal via colostrum.^23^ One mare, immune after re‐inoculation with *A. phagocytophilum* after recovery from initial experimental infection, produced foals during 3 consecutive breeding seasons. Each foal was challenged once with an inoculation of *A. phagocytophilum* at ages 15 days, 2 months, or 3 months respectively. No clinical signs or inclusion bodies were detected in the 2‐month‐old foal. Inclusion bodies were found in 0.5% to 1.0% of neutrophils of the 15‐day old foal, and the 3‐month‐old foal was febrile for 4 days with inclusion bodies on blood smear.^23^

In cattle, maternal antibodies against *Anaplasma marginale*, an organism closely related to *A. phagocytophilum*, have a half‐life of 5.54 weeks and most calves are seronegative by 21 weeks of age.^21^ Only 2/22 (9%) foals in the current study maintained a positive titer at 3 months and only 1 of those foals maintained a positive titer at 6 months. Three foals seroconverted between 3 and 6 months of age, all of which had positive titers at the day‐old examination and negative titers at 3 months of age. Two of these foals developed fevers between 3 and 6 months of age. These findings suggest natural exposure to *A. phagocytophilum* occurred before 6 months of age, and potentially resulted in clinical EGA in these foals.

One limitation of the current study includes the lack of umbilical cord blood samples or samples collected from foals prior to the ingestion of colostrum. Inclusion of a pre‐nursing sample to confirm colostral transfer of antibody has been performed in previous studies investigating equine passive immunity.^24^, ^25^ Others, however, have successfully demonstrated equine maternal antibody transfer against different diseases including *Rhodococcus equi* without inclusion of a pre‐nursing sample.^26^ Pre‐nursing samples were excluded in the current study due to the feasibility of on‐farm sampling at the time of parturition.

Transfer of naturally acquired specific immunity against *Anaplasma phagocytophilum* via colostrum occurs in foals born to seropositive mares and declines significantly by 3 months of age. The role of such immunity in protecting foals from developing clinical EGA remains unclear.

## CONFLICT OF INTEREST STATEMENT

The authors declare no conflict of interest.

## OFF‐LABEL ANTIMICROBIAL DECLARATION

Authors declare no off‐label use of antimicrobials.

## INSTITUTIONAL ANIMAL CARE AND USE COMMITTEE (IACUC) OR OTHER APPROVAL DECLARATION

Approved by the University of Pennsylvania IACUC (protocol # 806869).

## HUMAN ETHICS APPROVAL DECLARATION

Authors declare human ethics approval was not needed for this study.

## Supporting information


**TABLE S1.** Immunofluorescence Antibody Test (IFAT) antibody titers of 22 mare‐foal pairs to *Anaplasma phagocytophilum* in Southeastern Pennsylvania and Northern Maryland
