# Supplementary material for: Transfer of naturally acquired specific passive immunity against Anaplasma phagocytophilum in foals in Southeastern Pennsylvania and Northern Maryland
Source: J Vet Intern Med. 2023 Jul 28;37(5):1889–92. doi: 10.1111/jvim.16812 (PMC10472987; doi:10.1111/jvim.16812)
Supplement: Supplementary file 1 — TABLE S1. Immunofluorescence Antibody Test (IFAT) antibody titers of 22 mare‐foal pairs to Anaplasma phagocytophilum in Southeastern Pennsylvania and Northern Maryland [file JVIM-37-1889-s001.pdf]

Supplemental Table 1: Immunofluorescence Antibody Test (IFAT) antibody titers of 22 mare-foal pairs to *Anaplasma phagocytophilum* in Southeastern Pennsylvania and Northern Maryland

Mare ID	IFAT antibody titer -pre-foaling	IFAT antibody titer -24 hours of foaling	Foal ID	IFAT antibody titer -24 hours of foaling	IFAT antibody titer -3 months of age	IFAT antibody titer -6 months of age
Mare 1	1:200	1:100	Foal 1	Missing	Negative	Negative
Mare 2	1:200	1:100	Foal 2	1:100	Negative	Negative
Mare 3	1:100	1:100	Foal 3	1:100	Negative	Negative
Mare 4	1:200	1:100	Foal 4	1:100	Negative	Negative
Mare 5	1:100	1:100	Foal 5	1:100	Negative	1:100
Mare 6	1:100	1:100	Foal 6	1:50	Negative	1:50
Mare 7	1:50	1:50	Foal 7	1:50	Negative	Negative
Mare 8	1:50	1:50	Foal 8	1:50	Negative	Negative
Mare 9	1:100	1:200	Foal 9	Negative	Negative	Negative
Mare 10	1:100	1:50	Foal 10	1:100	Negative	Negative
Mare 11	1:100	1:400	Foal 11	1:400	Negative	Negative
Mare 12	1:200	1:200	Foal 12	1:200	Negative	Negative
Mare 13	1:200	1:100	Foal 13	1:100	1:50	1:50
Mare 14	1:200	1:200	Foal 14	1:50	Negative	Negative
Mare 15	1:50	1:100	Foal 15	Negative	Negative	Negative
Mare 16	1:50	1:100	Foal 16	Negative	Negative	Negative
Mare 17	Negative	Negative	Foal 17	Negative	Negative	Negative
Mare 18	Negative	1:50	Foal 18	1:50	Negative	Negative
Mare 19	1:100	1:200	Foal 19	1:200	Negative	Negative
Mare 20	1:50	1:100	Foal 20	1:100	Negative	1:50
Mare 21	Negative	Negative	Foal 21	Negative	Negative	Negative
Mare 22	1:100	1:200	Foal 22	1:100	1:50	Negative
